# TNF-α is associated with loss of lean body mass only in already cachectic COPD patients

**DOI:** 10.1186/1465-9921-13-48

**Published:** 2012-06-18

**Authors:** Tomas ML Eagan, Esteban C Gabazza, Corina D’Alessandro-Gabazza, Paloma Gil-Bernabe, Shinya Aoki, Jon A Hardie, Per S Bakke, Peter D Wagner

**Affiliations:** 1 Department of Thoracic Medicine, Haukeland University Hospital, N-5021, Bergen, Norway; 2 Section of Pulmonary Medicine, Institute of Medicine, University of Bergen, N-5021, Bergen, Norway; 3 Division of Physiology, University of California San Diego, 9500 Gilman Drive, La Jolla, San Diego, CA, 92093, USA; 4 Department of Immunology, Mie University School of Medicine, Edobashi 2-174, Tsu city, Mie, 514-8507, Japan; 5 Institue of Medicine, Haraldsplass Diakonale Sykehus, University of Bergen, N-5009, Bergen, Norway

**Keywords:** Inflammation, TNF-α, COPD, Cachexia

## Abstract

**Background:**

Systemic inflammation may contribute to cachexia in patients with chronic obstructive pulmonary disease (COPD). In this longitudinal study we assessed the association between circulating C-reactive protein (CRP), tumor necrosis factor (TNF)-α, interleukin (IL)-1ß, and IL-6 levels and subsequent loss of fat free mass and fat mass in more than 400 COPD patients over three years.

**Methods:**

The patients, aged 40–76, GOLD stage II-IV, were enrolled in 2006/07, and followed annually. Fat free mass and fat mass indexes (FFMI & FMI) were calculated using bioelectrical impedance, and CRP, TNF-α, IL-1ß, and IL-6 were measured using enzyme immunoassays. Associations with mean change in FFMI and FMI of the four inflammatory plasma markers, sex, age, smoking, FEV_1_, inhaled steroids, arterial hypoxemia, and Charlson comorbidity score were analyzed with linear mixed models.

**Results:**

At baseline, only CRP was significantly (but weakly) associated with FFMI (r = 0.18, p < 0.01) and FMI (r = 0.27, p < 0.01). Univariately, higher age, lower FEV_1_, and use of beta2-agonists were the only significant predictors of decline in FFMI, whereas smoking, hypoxemia, Charlson score, and use of inhaled steroids predicted increased loss in FMI. Multivariately, high levels of TNF-α (but not CRP, IL-1ß or IL-6) significantly predicted loss of FFMI, however only in patients with established cachexia at entry.

**Conclusion:**

This study does not support the hypothesis that systemic inflammation is the cause of accelerated loss of fat free mass in COPD patients, but suggests a role for TNF-α in already cachectic COPD patients.

## Background

Chronic obstructive pulmonary disease (COPD) afflicts approximately 9-10% of adults over 40 years of age [1], and is a source of considerable comorbidity and mortality to those afflicted [2,3]. A common complication in COPD is loss of skeletal muscle mass. Roughly 25% of COPD patients will develop cachexia [4], which by itself is associated with increased mortality [5].

Patients with COPD exhibit chronic inflammation in the airways, and several studies have shown higher systemic levels of inflammatory markers in patients with COPD compared with subjects without COPD [6-9]. Several authors have suggested that this systemic inflammation is a causal factor in the development of complications and comorbidities in patients with COPD, including the development of cachexia [10-12].

It is still controversial whether the increased systemic levels of inflammation represent a chronic, systemic inflammatory process or reflect spillover into the systemic bloodstream of inflammatory byproducts from the lungs without consequences [13]. Regardless of the source of the systemic inflammatory markers, increased levels could be a factor in the development of extra-pulmonary manifestations in COPD. This is a particularly attractive theory regarding Tumor Necrosis Factor alpha (TNF-α) and the development of cachexia in COPD. TNF-α, originally termed cachexin, is produced by a variety of immune cells, and is together with Interleukin-1ß and −6 (IL-1ß and IL-6) instrumental in the induction of the acute-phase response, including production of C-Reactive Protein (CRP). It has long been thought that prolonged exposure to TNF-α contributes to cachexia in cancer patients [14]. Thus, it would seem reasonable that higher systemic levels of CRP, TNF-α, IL-1ß, and IL-6 could be contributing factors to cachexia also in COPD patients.

So far, only cross-sectional studies have been published on levels of these inflammatory markers and body composition in patients with COPD, with some studies finding higher TNF-α in underweight COPD patients [15-18], whereas other studies have not [19-22].

The aim of the current study was to examine prospectively the relationship between the systemic inflammatory markers CRP, TNF-α, IL-1ß and IL-6 and loss of fat free mass over a 3-year time period, using data from the Bergen COPD Cohort Study in Western Norway. We hypothesized that patients with high levels of these molecules on entry would have a higher rate of decline in free fat mass over the next three years.

## Methods

### Study population

The study sampling and data collection in the baseline phase of the Bergen COPD Cohort Study have been published previously [7]. Briefly, in 2006/07, 426 COPD patients were enrolled, aged 40–76 years, with a clinical diagnosis of COPD, GOLD stage II or worse, and a smoking history of more than 10 pack-years. All patients received written and oral information prior to participation, and signed informed consent. The regional ethical committee (REK-Vest) approved the study.

All patients were examined at baseline, and attempted followed-up yearly. 388 patients (91%) participated at the one-year visit, 374 (88%) at the two-year visit, and 360 (85%) at the three-year visit. Eleven patients who used oral steroids were excluded from the study sample for the current study. For seven patients we lacked valid plasma samples, thus the initial study sample consisted of 408 patients.

### Data collection

A physician performed a structured interview, measured arterial blood gases, and provided health care as needed. Bioelectrical impedance was measured with a Bodystat 1500 (Bodystat Ltd, Douglas, Isle of Man, UK), and all patients were fasting prior to the visits. The reliability of bioelectrical impedance measurements has been previously described [23]. The fat free mass index (FFMI) and fat mass index (FMI) was calculated as the fat free mass (kg) or fat mass (kg) respectively, divided by the square of height (m^2^). A reliability study was performed for quality control of the impedance measurements. Ten COPD patients and 10 healthy volunteers were measured 10 times each within one hour. The coefficient of variance for FFMI was 0.47 for the patients and 0.54 for the healthy controls, and for FMI 1.14 for the patients and 2.09 for the controls. Cachexia was defined as having a FFMI less than 14 kg/m^2^ for women and less than 17 kg/m^2^ for men [4], which corresponds to the lower 95% confidence limit in a normal population [24].

For the current study, available plasma samples from baseline (n = 408) and the one-year (n = 382) visits were analyzed. The outcomes were change in FFMI and FMI, measured at baseline (n = 405) and the one- (n = 369), two- (n = 336), and three- (n = 339) year visits.

### Laboratory measurements

Plasma samples were drawn into pyrogen-free blood collection tubes with EDTA and centrifuged within 30 minutes at 2150 X g for 15 minutes at 4°C. All samples were stored at −80°C.

TNF-α, IL-1ß and IL-6 were measured using enzyme immunoassays (EIA) kits from BD Biosciences Pharmingen (San Diego, CA) and CRP using a high-sensitivity EIA kit from R&D Systems (Minneapolis, MN). All samples from one patient were analyzed on the same ELISA plate for each marker to reduce intra-subject inter-assay variations. All measurements were performed in duplicates and the averaged values were used.The detection limit for IL-1ß, IL-6 and TNF-α was 0.5 pg/mL, whereas for CRP it was 0.010 ng/ml and values below were defined as 0. The intra-assay and inter-assay coefficient of variations were <10% for all parameters.

### Statistical analyses

For the four markers the distribution was left-skewed. For the correlation analyses between all markers and the body composition indices FFMI and FMI, Spearman’s rank correlation tests were used. The levels of the cytokines were generally low, with many patients having levels below the detection limit for each of the visits. Therefore, in addition to looking at the baseline levels of each inflammatory marker we examined patients who had a measured plasma level above the 75 percentile of each marker both at entry and year 1 (“sustained high”), compared with patients who did not.

To properly adjust for the correlation between the repeated measurements of the outcome variables FFMI and FMI, advanced longitudinal analyses are necessary. Currently, the two most used methods are linear mixed models and generalized estimating equations (GEE) [25]. We applied linear mixed models using the xtmixed comand in Stata, with an unstructured variance-covariance structure. However, all analyses were also performed with generalized estimating equations (GEE), with an exchangeable correlations structure, for comparison.

#### Univariate analyses

For the univariate analyses, for both the outcomes FFMI and FMI we calculated one linear mixed model for each predictor variable, which included the variable in question, the effect of time (in years, coded as 0,1,2 or 3), and the interaction term of variable * time. To obtain interpretable intercepts, continuous predictor variables were centered; the inflammatory markers around their mean value, age around 60 years, and FEV_1_ around 50% of predicted.

For each model the coefficient of the variable in question thus represents the intercept, i.e. the effect of that variable at baseline, and the coefficient of time represents the effect of time. The real interest lies in the coefficients of the interaction term, as this specifies the effect of change in the outcome over time. With our coding of time as 0,1,2 and 3, the coefficients thus represents yearly change in mean FFMI or FMI.

#### Multivariable analyses

The main outcome of interest in this study was whether the plasma levels of the inflammatory markers predicted change in FFMI or FMI. Thus, the most important consideration with regards to the other variables like sex, age, smoking and FEV_1_ was to ensure adequate adjustment for possible confounding variables. For the multivariable analyses, we chose the following modeling strategy: We fitted one model for each inflammatory marker for each outcome, in which each model included the effect of sex, age, cachexia, smoking, FEV_1_ in percent predicted, hypoxemia, Charlson Index, exacerbations the last year prior to inclusion, use of inhaled steroids, use of long-acting ß2agonists (LABA), and use of theophylline. Since several previous cross-sectional studies have shown an association between cachexia and the levels of some of these inflammatory markers, we tested for the interaction between cachexia and the marker in question for each model, with a significance level set at 0.05. All analyses were performed with Stata 12.1.

## Results

The baseline characteristics of the cohort are presented in Table 1. About half were at GOLD stage II, average age was a little more than 60 years, and more than two thirds used inhaled steroids at entry. Twenty-nine percent were cachectic at entry to the study by the criteria we used [4]. Patients lost to follow-up were older, had more advanced COPD with more frequent exacerbations, and with more comorbidities (Table 1). Among male patients, FFMI was lower among patients lost to follow-up (p = 0.02, Table 1). The baseline levels of the four markers were not different between patients with complete follow-up and patients lost to follow-up. 

**Table 1:** Baseline characteristics of the study sample and comparison with patients lost to follow-up

	**Women (n = 159)**	**Men (n = 249)**	**Lost to follow-up (n = 69)**	p**
*Age, mean years (SD)*	62.3 (6.7)	64.2 (7.0)	66.8 (5.9)	<0.01
FFMI, mean kg/m2 (SD)	14.8 (2.4)	18.4 (3.0)	-	
FMI, mean kg/m2 (SD)	10.1 (4.3)	7.3 (2.5)	-	
% cachectic*	31.0	27.5	37.3	0.10
*Smoking habits, n(%)*				0.32
ex	88 (55.3)	144 (57.8)	43 (62.3)	
current	71 (44.7)	105 (42.2)	26 (37.7)	
*GOLD stage*				<0.01
II	82 (51.6)	111 (44.6)	22 (31.9)	
III	66 (41.5)	105 (42.2)	31 (44.9)	
IV	11 (6.9)	33 (13.2)	16 (23.2)	
*Hypoxemia, n(%)*				<0.01
PaO2 > 9 kPa	84 (61.3)	150 (64.4)	26 (44.1)	
PaO2 8–9 kPa	33 (24.1)	56 (24.0)	17 (28.8)	
PaO2 < 8 kPa	20 (14.6)	27 (11.6)	16 (27.1)	
*Exacerbations last year, n(%)*				0.01
0-1	126 (79.3)	214 (85.9)	50 (72.5)	
2+	33 (20.7)	35 (14.1)	19 (27.5)	
Charlson Comorbidity Index				<0.01
1	101 (63.5)	130 (52.2)	30 (43.5)	
2	40 (25.2)	59 (23.7)	15 (21.7)	
3	13 (8.2)	36 (14.5)	12 (17.4)	
4+	5 (3.1)	24 (9.6)	12 (17.4)	
*Use of inhaled steroids*				0.19
No	40 (25.2)	88 (35.3)	17 (24.6)	
Yes	119 (74.8)	161 (64.7)	52 (75.4)	
*Use of long-acting beta2 agonists*				0.07
No	33 (20.8)	80 (32.1)	13 (18.8)	
Yes	126 (79.2)	169 (67.9)	56 (81.2)	
*Use of theophyllin*				0.02
No	144 (90.6)	228 (91.6)	58 (84.1)	
Yes	15 (9.4)	21 (8.4)	11 (15.9)	
CRP, mean ug/mL (SD)	2.4 (3.1)	2.4 (2.7)	2.5 (2.5)	0.35
TNF-α mean pg/mL (SD)	1.8 (3.4)	1.8 (3.3)	1.2 (2.5)	0.12
IL-1ß, mean pg/mL (SD)	0.8 (2.0)	1.0 (2.2)	1.0 (2.1)	0.77
IL-6, mean pg/mL (SD)	2.2 (6.8)	2.2 (5.5)	1.4 (2.7)	0.32

*defined as FFMI < 14 kg/m2 for women and <17 kg/m2 for men.

**for difference between baseline and lost to follow up; chi square for categorical variables and Kruskal Wallis test for continuous variables.

**Figure 1:**
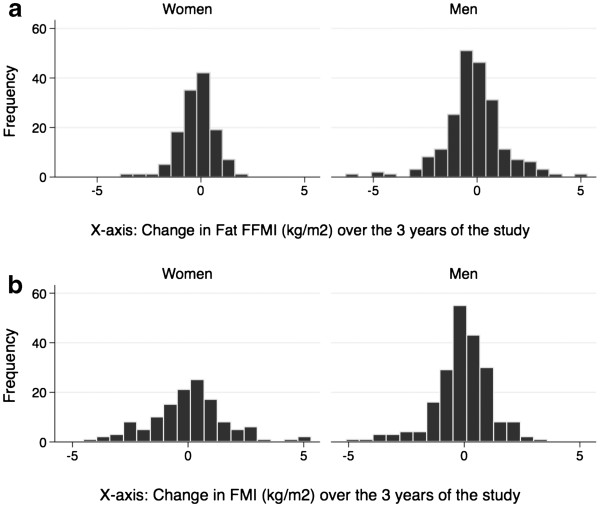
Distribution of change in fat free mass index (FFMI) and fat mass index (FFMI) (kg/m2) over the 3 years of the study.

The distribution of change in FFMI and FMI over the three years of the study is shown in Figure 1.

### Univariate analyses between independent variables and FFMI or FMI

A correlation matrix of the four inflammatory markers measured on entry (year 0) and at second visit (year 1), as well as FFMI and FMI measured at these two visits, is presented in Table 2. There were significant correlations between the levels of each marker between the first and second visit, strongest for CRP (correlation coefficient 0.75). There were also significant correlations between the different cytokines, but weaker than the inter-visit correlation for each marker. The only inflammatory marker that consistently correlated with FFMI and FMI at the first and second visit was CRP.

**Table 2:** Correlation coefficients for univariate relationships between plasma inflammatory markers at baseline (year 0) and first follow-up visit (year 1), and FFMI and FMI at those two visits

	**CRP**,**year 0**	**CRP, year 1**	**TNF-α year 0**	**TNFα year 1**	**IL-1ß, year 0**	**IL-1ß, year 1**	**IL-6, year 0**	**IL-6, year 1**	**FFMI, year 0**	**FFMI, year 1**	**FMI, year 0**	**FMI, year 1**
*CRP, year 0*	1											
*CRP, year 1*	0.75**	1										
TNF-α year 0	−0.05	−0.10	1									
TNF-α year 1	−0.02	−0.02	0.49**	1								
IL-1ß, year 0	0.10	0.06	0.30**	0.20**	1							
IL-1ß, year 1	0.05	0.08	0.21**	0.17**	0.47**	1						
IL-6, year 0	0.17**	0.19**	0.27**	0.22**	0.24**	0.20**	1					
IL-6, year 1	0.11*	0.15**	0.17**	0.36**	0.08	0.15**	0.43**	1				
FFMI, year 0	0.18**	0.13*	−0.05	0.06	0.03	−0.04	0.08	0.10	1			
FFMI, year 1	0.19**	0.15**	−0.05	−0.01	0.08	0.00	0.10	0.03	0.95**	1		
FMI, year 0	0.27**	0.16**	−0.04	0.06	0.05	−0.01	0.02	0.10*	0.25**	0.30**	1	
FMI, year 1	0.27**	0.15**	−0.01	0.01	0.06	−0.02	0.01	0.03	0.26**	0.26**	0.93**	1

* p < 0.05, ** p < 0.01; Spearman rank correlation coefficients.

The unadjusted estimated yearly change in FFMI and FMI for each inflammatory marker is shown in Table 3. For example, in a patient with a measured value of 2.4 ug CRP, the yearly decline in FFMI would be 0.0732 kg/m2. For a patient with a measured level of CRP equal to 3.4 ug, the yearly decline in FFMI would be (0.0732-0.0121) 0.0611 kg/m2, whereas in a patient with measured level of CRP 1.4, the yearly decline in FFMI would be (0.0732 + 0.0121) 0.0853 kg/m2.

**Table 3:** Unadjusted yearly change in fat free mass index (FFMI) and fat mass index (FMI) for the four infalammatory markers, estimated by linear mixed models

	**Yearly change in FFMI**	**p-value**	**Yearly change in FMI**	**p-value**
*CRP*				
at CRP = 2.4	−0.0732		−0.0195	
per 1 ug/mL increase	0.0121	0.10	−0.0041	0.63
*Sustained high CRP at year 0 and 1*				
No	−0.0828		0.0037	
Yes	0.0320	0.046	−0.1421	0.02
*TNF-α*				
at TNF-α = 1.8	−0.0597		−0.0236	
per 1 pg/mL increase	−0.0107	0.09	0.0020	0.77
*Sustained high TNF-α at year 0 and 1*				
No	−0.0488		−0.0283	
Yes	−0.1621	0.07	0.0366	0.36
*IL-1ß*				
at IL-1ß = 0.93	−0.0611		−0.0234	
per 1 pg/mL increase	0.0144	0.16	−0.0026	0.82
*Sustained high IL-1ß at year 0 and 1*				
No	−0.0723		−0.0216	
Yes	−0.0167	0.36	−0.0083	0.85
*IL-6*				
at IL-6 = 2.8	−0.0613		−0.0234	
per 1 pg/mL increase	0.0023	0.10	−0.0003	0.87
*Sustained high IL-6 at year 0 and 1*				
No	−0.0700		−0.0150	
Yes	−0.0251	0.49	−0.0558	0.58

For neither FFMI nor FMI were the entry inflammatory marker levels statistically significant predictors of increased decline. However, in patients with a sustained high level of CRP, there was a significant increase in FFMI and decrease in FMI over three years.

Higher age, lower FEV_1_, and use of long-acting beta2-agonists were the only significant predictors of an increased decline in FFMI in the univariate analyses. For decline in FMI, significant predictors were smoking history, arterial oxygen tension (PaO_2_) less than 8 kPa, the presence of comorbidities, use of inhaled steroids (Table 4). Exacerbation history the last year before entry to the study, did not predict change in either FFMI or FMI (Table 4).

**Table 4:** Unadjusted yearly change in fat free mass index (FFMI) and fat mass index (FMI) for each baseline predictor variable, estimated bylinear mixed models

**Baseline variables**	**Yearly change in FFMI**	**p-value**	**Yearly change in FMI**	**p-value**
*Sex*				
Women	−0.0613		0.01599	
Men	−0.0662	0.91	−0.04131	0.24
*Age*				
at age 60	−0.0377		−0.0024	
per 10 yrs increase	−0.0867	0.01	−0.0584	0.10
*Smoking*				
Ex	−0.0772		−0.0762	
Current	−0.0477	0.49	0.0531	0.01
*GOLD stage*				
II	0.0024		0.0032	
III	−0.1151	0.01	−0.0333	0.47
IV	−0.1861	0.01	−0.0921	0.27
*FEV1*				
at 50% of predicted	−0.0624		−0.0197	
per 10% decrease in predicted value	−0.0477	0.00	−0.0227	0.18
*Hypoxemia*				
PaO2 > 9 kPa	−0.0664		0.0006	
PaO2 8–9 kPa	−0.0506	0.77	0.0270	0.65
PaO2 < 8 kPa	−0.1052	0.61	−0.2695	0.00
*Exacerbations last year*				
0-1	−0.0618		−0.0115	
2+	−0.0764	0.81	−0.0638	0.43
*Charlson Comorbidity Score*				
I	−0.0768		0.0378	
II	−0.0652	0.82	−0.0737	0.05
III	0.0333	0.11	−0.1616	0.01
IV+	−0.1231	0.62	−0.0962	0.20
*Cachexia*				
No	−0.0495		−0.0255	
Yes	−0.0983	0.31	−0.0089	0.76
*Using inhaled steroids*				
No	−0.0243		0.0610	
Yes	−0.0828	0.20	−0.0574	0.02
*Use of long-acting beta2 agonists*				
No	−0.0101		0.0696	
Yes	−0.0862	0.01	−0.0557	0.02
*Use of theophyllin*				
No	−0.0605		−0.0086	
Yes	−0.1097	0.54	−0.1421	0.14

Estimation with GEE gave reasonable accordance with the results found with the linear mixed models, where all coefficients where almost similar, but the confidence intervals sometimes narrower (data not shown).

### Multivariable analyses

After adjustment for all covariables, lower CRP and lower IL-6 at baseline was significantly associated with an increased decline in FFMI (Table 5). For CRP this also held up when comparing patients with sustained high levels to patients without, where the fastest decline in FFMI was seen among patients without sustained high levels of CRP.

**Table 5:** Adjusted* yearly change in fat free mass index (FFMI) and fat mass index (FMI) for plasma CRP, TNF-α, IL-1ß and IL-6, estimated by linear mixed models with random effects

	**Yearly change in FFMI**	**p-value**	**Yearly change in FMI**	**p-value**
*CRP at baseline*				
at CRP = 2.4	−0.0622		0.1355	
per 1 ug/mL increase	0.0221	0.01	−0.0066	0.48
*Sustained high CRP at year 0 and 1*				
No	−0.0874		0.1494	
Yes	0.0656	0.01	0.0061	0.03
*TNF-α at baseline*				
at TNF-α = 1.8	−0.0531		0.1303	
per 1 pg/mL increase	−0.0098	0.13	0.0004	0.96
*Sustained high TNF-α at year 0 and 1*				
No	−0.0643		0.1287	
Yes	−0.2098	0.03	0.1366	0.91
*IL-1ß at baseline*				
at IL-1ß = 0.93	−0.0424		0.1300	
per 1 pg/mL increase	0.0147	0.16	0.0009	0.93
*Sustained high IL-1ß at year 0 and 1*				
No	−0.0685		0.1257	
Yes	−0.0341	0.58	0.1548	0.67
*IL-6 at baseline*				
at IL-6 = 2.8	−0.0414		0.1302	
per 1 pg/mL increase	0.0034	0.02	0.0004	0.79
*Sustained high IL-6 at year 0 and 1*				
No	−0.0676		0.1291	
Yes	0.0231	0.18	0.1184	0.88

* Adjusted for the effect of change by the interaction between yearly change and each of the baseline variables sex, age, cachexia, FEV1, smoking, hypoxemia, Charlson Index, number of exacerbations the last year, use of inhaled steroids, long acting beta2agonists, and use of theophylline.

Patients with a sustained high level of TNF-α (TNF-α > 2.2 pg/mL, n = 54) at both year 0 and year 1, had a significantly greater decline in FFMI compared with patients without sustained high TNF-α (Table 5).

The only significant relationship between levels of the inflammatory markers and change in FMI over time was in patients with sustained high levels of CRP who had a smaller increase in FMI than patients without sustained high levels of CRP. (Table 5).

Cachexia at baseline was not associated with accelerated decline in either FFMI or FMI, neither in the univariate analyses (Table 4), nor when included in the multivariable analyses (data not shown).

**Figure 2:**
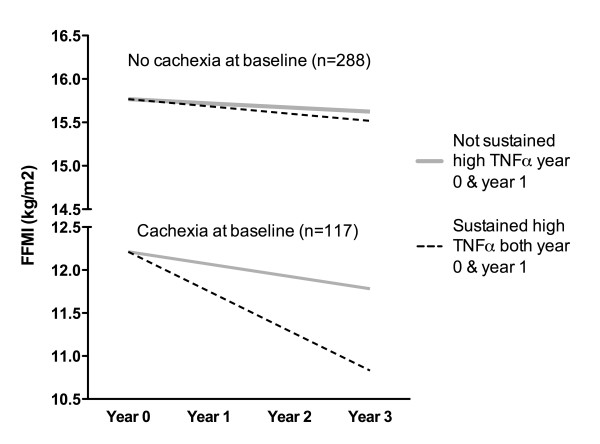
The estimated change in FFMI based on the regression coefficients from the interaction (p = 0.013) between the effects of plasma levels of TNF-α on change in fat free mass in cachectic and non-cachectic COPD patients.

For TNF-α there was a significant interaction with cachexia at baseline in patients with sustained high TNF-α at year 0 and year 1 (p = 0.001). This is demonstrated in Figure 2, which displays the estimated yearly change in FFMI for patients with and without a sustained high level of TNF-α and with and without cachexia at baseline, calculated from the coefficients from the multivariable regression analysis. Figure 2 shows that only for COPD patients who were cachectic did the slopes differ significantly between patients with different levels of TNF-α. Specifically, in already cachectic patients, sustained high TNF-α levels were associated with greater decline in FFMI than for those without sustained high TNF-α. However, in non-cachectic patients, sustained high TNF-α was not associated with greater decline in FFMI.

## Discussion

This is to our knowledge the first longitudinal study of the relationships between the systemic inflammatory markers CRP, TNF-α, IL-1ß and IL-6 and the change in body composition over time in patients with COPD. The main finding from this study was that, over 3 years, sustained high TNF-α levels were associated with an accelerated decline in fat free mass, however, an interaction analysis suggested this may be only in COPD patients who were already cachectic at entry into the study.

### Inflammation and loss of FFMI

COPD is a chronic inflammatory disorder, and several studies have found increased levels of TNF-α, as well as CRP and IL-6 both in bronchoalveolar lavage (BAL) [26] and in the systemic circulation [26,27]. More importantly, some studies have shown higher systemic levels of TNF-α in COPD patients with cachexia compared with patients without cachexia [15-17]. However, the measured levels of TNF-α in BAL and blood correlate poorly [13], and some studies have not found higher levels of TNF-α in COPD patients with cachexia compared with patients without cachexia [19,20,22].

In the current study, we have not compared the levels of the markers to non-COPD subjects. Compared with levels of inflammatory markers measured in other normal populations [28-30], our levels were higher. However, we caution against over-interpretation of this, since methodology and populations differed.

The measured levels of cytokines in the peripheral circulation are usually quite low, partly due to the typical ‘burst secretion’ of the cytokines, and partly due to the fact that there are many available soluble proteins to which the cytokines can bind. Thus, to observe the potential effects of a sustained increase of the pro-inflammatory cytokines in the systemic circulation, a sufficient patient sample is necessary, which has not always been the case with studies published so far. We studied over 400 patients, 29% of whom were cachectic.

### Longitudinal versus cross-sectional studies

Arguably, the most important missing pieces in the literature to date are longitudinal studies on humans. Although cross-sectional studies may indicate an association between levels of an inflammatory marker and body composition, the rate of change in FFMI and FMI can only be assessed with longitudinal data. We have previously shown from a cross-sectional study on this cohort that CRP was markedly increased in COPD patients compared with subjects without COPD [7]. In addition, we then found that fat mass, more than fat free mass, was associated with elevated levels of CRP in COPD patients [31].

In the current 3-year longitudinal study, we expand on prior knowledge by showing that it was COPD patients without sustained high levels of CRP that had the largest decline in FFMI and largest increase in FMI. Further we showed that COPD patients with higher systemic levels of IL-1ß and IL-6 in general did not have a subsequently increased decline in fat free mass or increase in fat mass. Only one marker, TNF- α, and only in patients with a high level both at year 0 and year 1, predicted an increased decline in FFMI. However, the interaction analyses suggested that COPD patients with the same high TNF-α levels who were not cachectic on entry did not lose FFMI faster than their non-cachectic counterparts with lower TNF-α.

### Interpretation of the findings

If we accept the current findings, the question remains, what do they mean?

There are several possible interpretations: First, inflammation may be a consequence rather than a cause of initial loss of fat free mass, but may contribute to further loss of fat free mass once the cachectic process has started. Second, only a subgroup of patients respond to high TNF-α levels by loss of FFMI, presumably due to genetic and/or environmental differences between subgroups. Third, the patients who were cachectic at entry may have had a longer exposure to inflammation before entering the study, implying that the non-cachectic patients with high TNF-α levels will develop cachexia if followed longer. It would take and even bigger and far longer study to distinguish among these possibilities – one in which prior to starting to smoke, a young cohort was followed until age 60–70 with similar measurements. In that way, pre-tobacco influences on cytokine levels could be established, time to develop COPD would be recorded, and temporal relationships between body composition and cytokine profiles established.

These possibilities could have therapeutic consequences, since a TNF-α blocking agent presumably would not have an effect before cachexia was already developed if the first explanation was correct. Very few studies to date have assessed anti- TNF-α treatment on cachexia, and only in patients with cancer [32] or arthritis [33]. In neither of the studies to date have the results been convincingly in favor of anti- TNF-α treatment. Although there are no specific trials to date on anti- TNF-α treatment for cachexia in COPD, interesting circumstantial evidence is available from a multi-centre trial of 220 COPD patients in North-America, in which infliximab was tested against placebo [34]. In patients who were cachectic and received infliximab 6-minute walking distance improved significantly after 24 weeks, whereas patients who were not cachectic did not benefit from the drug, supporting our findings that TNF-α may be of consequence only in already cachectic COPD patients.

Regarding change in FMI, baseline variables daily smoking, use of inhaled steroids, and being hypoxemic were associated with a larger loss of FMI, even in the multivariable model (data not shown). Current smoking is associated with a suppression of appetite, whereas use of inhaled steroids could be a marker for disease severity. Patients with respiratory failure may exert themselves more at rest, thus burning more fat. However, we caution against over-interpretation, as the analyses did not take into account change in smoking habits, PaO_2_ or medication use throughout the study. The main research interest was whether plasma levels of the inflammatory markers were predictive of subsequent change in body composition, including changes in fat mass, and the importance of the co-variables was to make sure we had adequately adjusted for confounders.

### Measurement considerations

The results of the current study are unlikely to be due only to the inherent methodological difficulties. First, the levels of the cytokines measured in the COPD patients were about the same as other authors have reported in recent studies [19,35]. Standard high-sensitivity ELISA kits were used, all samples were analyzed in duplicate, and all samples from the same subject were analyzed on the same plate. Second, there was a positive, albeit weak, correlation between the three cytokines at each year, as expected. Third, we separately analyzed the predictive value of a) the level of each of the cytokines at entry, and b) a sustained high level of the cytokine for the first year, with the same conclusion. Finally, we also analyzed the predictive value of the cytokines at entry as categorical variables (low, medium, high), and this also produced the same result (data not shown): TNF-α was the only cytokine associated with change in FFMI, and in already cachectic COPD patients only.

The other potentially important methodological issue is the measurement of change in FFMI and FMI. Over the three years of the study, the mean yearly change in both FFMI and FMI was not very large. However, even though overall mean change was small, the range of change was quite significant (Figure 1). This range enables analyses of whether change in one group is significantly different from change in another group.

Finally, the analyses of the socio-demographic and clinical variables demonstrated significant and reasonable predictors of change in lean body mass. Higher age and lower FEV_1_ were significantly related to faster decline in fat free mass, as expected.

In conclusion, this study did not find firm support for the systemic inflammation hypothesis in causing COPD cachexia, but raises the possibility that TNF-α contributes to further loss of FFMI in patients who are already cachectic, creating a vicious circle of ever increasing inflammation and loss of fat free mass.

## Competing interests

The authors declare that they have no competing interests.

## Authors’ contributions

TE participated in planning the study, data collection, the laboratory analyses, the statistical analyses, and the writing of the manuscript. EG participated in planning the study, the laboratory analyses, the statistical analyses, and the writing of the manuscript. CD participated in planning the study, the laboratory analyses, and the writing of the manuscript. PG participated in the laboratory analyses and the writing of the manuscript. SA participated in the laboratory analyses and the writing of the manuscript. JH participated in planning the study, data collection, and the writing of the manuscript. PB participated in planning the study, data collection, the statistical analyses, and the writing of the manuscript. PW participated in planning the study, the statistical analyses, and the writing of the manuscript. All authors read and approved the final manuscript.
